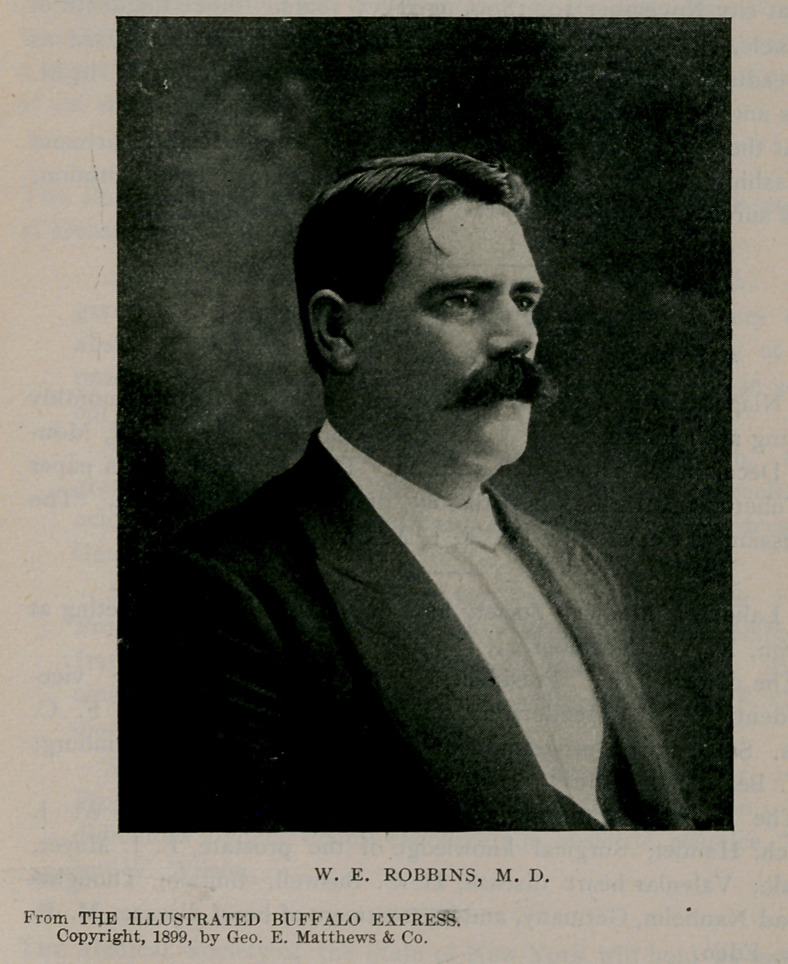# Dr. W. E. Robbins

**Published:** 1900-01

**Authors:** 


### OBITUARY.

Dr. W. E. Robbins, of Hamburg, N. Y., died at his residence December 5, 1899, aged 39 years. His sudden death was a great shock to the community where he lived. He had been ailing for several days previously, but no serious results were anticipated. His friends, the neighboring physicians, Drs. C. W. and B. S. Bourne, called to see him the morning of his death and while conversing with them he was taken with a convulsion and almost immediately expired.

Dr. Robbins was born in Iowa, on November 7, 1860; he came with his parents to Erie County when a small boy, the family settling in North Evans. There he lived until he entered the University of Buffalo, graduating with the class of '85. His standing in the profession had become high. He was conscientious in the discharge of his professional duties, and he acquired a large practice, especially in the southern part of the county. He was a member of the staff of the Erie County Hospital, and was a member of various medical societies. He had been health officer of the town of Hamburg and held a like position in the village at the time of his death.

W. E. ROBBINS, M. D.

From THE ILLUSTRATED BUFFALO EXPRESS.
Copyright, 1899, by Geo. E. Matthews & Co.

Dr. Robbins was identified with every public movement in his village and had the highest esteem of all its residents. He was a member of the Royal Arcanum and was senior warden of Fraternal Lodge, F & A. M., which body conducted the funeral services on Friday morning, December 8th, at 10 o'clock, preceded by a short address by the Rev. D. W. Jones. He is survived by a widow, two daughters, his father and four sisters.

## Figures and Tables

**Figure f1:**